# Supplementary material for: Horn-like space-coiling metamaterials toward simultaneous phase and amplitude modulation
Source: Nat Commun. 2018 Apr 10;9:1349. doi: 10.1038/s41467-018-03839-z (PMC5893611; doi:10.1038/s41467-018-03839-z)
Supplement: Supplementary file 1 — Supplementary Information [file 41467_2018_3839_MOESM1_ESM.pdf]

Supplementary Information: Horn-like space-coiling metamaterials toward simultaneous phase and amplitude modulation

Ghaffarivardavagh et al.

Supplementary Table 1. Geometrical features and associated transmission in metasurface

Unit cell	Metasurface function	Geometrical parameters				Resultant transmission coefficient					
						T-Reversal		Numerical		Analytical	
		N	*W* (*λ*)	d_1 (*λ*)	CR	Amp	Phase (degree)	Amp	Phase (degree)	Amp	Phase (degree)
1	Focusing	2	0.124	0.064	1	0.72	107	0.68	108	0.75	114
	Beam-splitter	10	0.026	0.009	1.25	0.37	-116	0.36	-115	0.39	-115
2	Focusing	5	0.028	0.041	1.25	0.72	60	0.77	61	0.76	60
	Beam-splitter	16	0.013	0.004	1.18	0.32	-124	0.31	-124	0.34	-124
3	Focusing	6	0.022	0.034	1.23	0.73	12	0.75	12	0.77	12
	Beam-splitter	9	0.038	0.003	1.48	0.31	-145	0.31	-146	0.34	-148
4	Focusing	13	0.004	0.025	1.05	0.79	-31	0.8	-30	0.82	-34
	Beam-splitter	9	0.035	0.004	1.43	0.39	-168	0.38	-166	0.41	-171
5	Focusing	8	0.004	0.058	1	0.79	-68	0.72	-65	0.72	-74
	Beam-splitter	16	0.010	0.007	1.15	0.54	-178	0.58	-178	0.59	179
6	Focusing	9	0.003	0.050	1.01	0.71	-108	0.72	-108	0.71	-116
	Beam-splitter	10	0.016	0.017	1.18	0.70	180	0.71	-178	0.74	177
7	Focusing	9	0.013	0.020	1.16	0.73	-153	0.72	-157	0.71	-156
	Beam-splitter	10	0.015	0.023	1.13	0.81	-179	0.82	-175	0.84	177
8	Focusing	9	0.015	0.026	1.1	0.87	173	0.86	169	0.89	180
	Beam-splitter	10	0.015	0.023	1.13	0.83	-177	0.82	-175	0.84	177
9	Focusing	2	0.103	0.086	1.2	0.91	149	0.93	150	0.96	153
	Beam-splitter	10	0.016	0.017	1.18	0.73	-176	0.71	-176	0.74	179
10	Focusing	3	0.064	0.064	1.25	0.83	124	0.86	124	0.89	126
	Beam-splitter	16	0.010	0.006	1.18	0.52	180	0.50	180	0.51	180
11	Focusing	4	0.042	0.051	1.24	0.78	94	0.8	92	0.81	92
	Beam-splitter	16	0.010	0.004	1.23	0.27	156	0.34	156	0.35	161
12	Focusing	5	0.023	0.058	1.1	0.85	68	0.87	69	0.84	67
	Beam-splitter	14	0.013	0.007	1.18	0.28	77	0.28	78	0.28	81
13	Focusing	6	0.007	0.073	1.01	0.92	56	0.95	56	0.91	49
	Beam-splitter	14	0.006	0.020	1.08	0.57	52	0.60	50	0.62	52
14	Focusing	6	0.009	0.067	1.04	0.92	52	0.94	53	0.91	48
	Beam-splitter	8	0.012	0.058	1.00	0.83	47	0.79	46	0.84	47
15	Focusing	5	0.028	0.067	1	0.9	51	0.93	50	0.86	54
	Beam-splitter	2	0.001	0.117	1.40	0.97	45	1.00	48	1.00	49

Supplementary Table 2. Experimental data points for the cases of with and without the metasurface

Experimental Data Without the Presence of the Metasurface										
	X1	*X2*	*X3*	*X4*	*X5*	*X6*	*X7*	*X8*	*X9*	*X10*
Y1	0.177+0.1i	0.395-0.02i	0.132-0.33i	-0.278-0.231i	-0.3+0.095i	-0.06+0.314i	0.212+0.218i	0.05-0.188i	-0.191-0.145i	0.09+0.274i
Y2	-0.076+0.253i	0.362+0.396i	0.433+0.036i	0.176-0.49i	-0.292-0.497i	-0.478+0.05i	-0.094+0.563i	0.314+0.215i	0.175-0.325i	-0.125-0.052i
Y3	-0.344-0.295i	-0.4+0.234i	-0.078+0.42i	0.375+0.175i	0.315-0.261i	-0.151-0.333i	-0.194+0.147i	0.237+0.207i	0.231-0.252i	-0.307-0.307i
Y4	0.183-0.499i	-0.423-0.34i	-0.533+0.135i	0.031+0.503i	0.542+0.298i	0.188-0.327i	-0.308-0.338i	-0.098+0.14i	0.277+0.181i	-0.001-0.179i
Y5	0.627+0.15i	0.29-0.345i	-0.279-0.49i	-0.362+0.036i	0.103+0.503i	0.231+0.009i	-0.2-0.438i	-0.341+0.034i	0.137+0.542i	0.365+0.108i
Y6	0.026+0.634i	0.566+0.445i	0.315-0.453i	-0.259-0.493i	-0.339+0.277i	0.164+0.402i	0.252-0.231i	-0.204-0.156i	-0.191+0.384i	0.264+0.149i
Y7	-0.568+0.101i	-0.13+0.739i	0.512+0.101i	0.149-0.518i	-0.439-0.007i	-0.009+0.428i	0.517-0.061i	0.092-0.323i	-0.452+0.066i	-0.109+0.096i
Y8	-0.446-0.644i	-0.781+0.389i	0.226+0.466i	0.432-0.314i	-0.384-0.166i	-0.288+0.319i	0.411-0.023i	0.185-0.392i	-0.442+0.039i	-0.099+0.197i
Y9	0.33-0.857i	-0.923-0.216i	0.019+0.534i	0.63-0.143i	-0.184-0.41i	-0.483+0.244i	0.214+0.165i	0.17-0.256i	-0.37+0.096i	0.072+0.36i
Y10	0.673-0.72i	-0.763-0.328i	-0.068+0.516i	0.752-0.132i	-0.183-0.484i	-0.484+0.255i	0.297+0.234i	0.3-0.245i	-0.402+0.003i	-0.031+0.282i
Y11	0.408-0.825i	-0.92-0.119i	-0.017+0.66i	0.649-0.113i	-0.271-0.432i	-0.4+0.212i	0.346+0.196i	0.308-0.365i	-0.493-0.107i	-0.223+0.138i
Y12	-0.145-0.807i	-0.872+0.202i	0.188+0.578i	0.442-0.229i	-0.413-0.26i	-0.31+0.392i	0.422+0.165i	0.169-0.306i	-0.469+0.021i	-0.065+0.217i
Y13	-0.519-0.323i	-0.315+0.55i	0.56+0.258i	0.285-0.624i	-0.472-0.156i	-0.21+0.555i	0.317+0.044i	-0.023-0.315i	-0.373+0.344i	0.181+0.37i
Y14	-0.372+0.547i	0.127+0.61i	0.544-0.196i	0.034-0.546i	-0.274+0.113i	0.151+0.373i	0.28-0.284i	-0.272-0.324i	-0.266+0.441i	0.103+0.278i
Y15	0.381+0.515i	0.33+0.071i	-0.149-0.466i	-0.429-0.056i	-0.051+0.533i	0.442+0.173i	0.144-0.502i	-0.282-0.239i	-0.087+0.392i	0.089-0.012i
Y16	0.643-0.474i	-0.03-0.442i	-0.559-0.176i	-0.288+0.277i	0.251+0.392i	0.247-0.004i	-0.169-0.25i	-0.149+0.174i	0.279+0.29i	0.136-0.2i
Y17	-0.127-0.548i	-0.264+0.041i	-0.111+0.36i	0.327+0.124i	0.32-0.287i	-0.223-0.312i	-0.432+0.101i	0.049+0.362i	0.458+0.06i	0.079-0.207i
Y18	-0.565+0.239i	-0.056+0.525i	0.301+0.395i	0.429-0.156i	0.18-0.482i	-0.325-0.184i	-0.269+0.213i	0.142+0.041i	0.204-0.226i	-0.147-0.063i
Y19	0.143+0.25i	0.377+0.021i	0.073-0.173i	-0.244-0.223i	-0.251+0.034i	0.343i	0.22+0.215i	0.111-0.283i	-0.176-0.36i	-0.205+0.154i

Experimental Data With the Presence of the Metasurface										
	X1	*X2*	*X3*	*X4*	*X5*	*X6*	*X7*	*X8*	*X9*	*X10*
Y1	-0.056+0.18i	-0.012+0.146i	0.089+0.111i	0.055-0.158i	-0.081-0.082i	-0.108+0.19i	0.05+0.074i	0.124-0.108i	-0.052-0.095i	-0.035+0.023i
Y2	-0.38-0.051i	-0.195+0.045i	0.141+0.208i	0.133-0.148i	-0.119-0.11i	-0.193+0.097i	0.049+0.056i	0.154+0.011i	0.014-0.052i	-0.051-0.006i
Y3	-0.389+0.471i	-0.287+0.203i	0.138+0.214i	0.227-0.085i	-0.072-0.07i	-0.093-0.007i	0.11-0.052i	0.04-0.002i	-0.163+0.056i	-0.033+0.011i
Y4	0.05+0.073i	0.034+0.137i	0.078-0.029i	0.084-0.108i	-0.05+0.072i	0.042+0.07i	0.144-0.142i	-0.074-0.055i	-0.212+0.163i	0.092+0.049i
Y5	0.092+0.048i	-0.019+0.011i	-0.085-0.247i	-0.097-0.155i	-0.11+0.148i	0.055+0.175i	0.153-0.14i	-0.077-0.123i	-0.172+0.171i	0.184+0.063i
Y6	-0.194+0.031i	-0.215-0.07i	-0.018-0.156i	0.123-0.035i	-0.123+0.153i	-0.08+0.12i	0.208-0.125i	-0.013-0.134i	-0.236+0.184i	0.102+0.04i
Y7	-0.259+0.188i	-0.472+0.076i	0.17+0.132i	0.455-0.03i	-0.179-0.019i	-0.273+0.021i	0.264-0.141i	0.092-0.115i	-0.318+0.168i	0.038+0.091i
Y8	-0.381+0.416i	-0.656+0.285i	0.326+0.346i	0.543-0.184i	-0.341-0.172i	-0.359+0.108i	0.429+0.001i	0.118-0.135i	-0.485+0.201i	0.109+0.169i
Y9	-0.555+0.096i	-0.654+0.233i	0.388+0.313i	0.49-0.438i	-0.616-0.305i	-0.344+0.454i	0.735+0.157i	0.091-0.368i	-0.757+0.076i	0.1+0.352i
Y10	-0.657+0.094i	-0.604+0.416i	0.489+0.27i	0.456-0.484i	-0.698-0.153i	-0.303+0.569i	0.96+0.09i	0.118-0.547i	-0.965-0.066i	0.01+0.438i
Y11	-0.361+0.382i	-0.586+0.417i	0.529+0.312i	0.533-0.328i	-0.545-0.138i	-0.301+0.413i	0.829+0.043i	0.141-0.425i	-0.82-0.037i	0.044+0.331i
Y12	-0.342+0.092i	-0.587+0.153i	0.281+0.26i	0.422-0.185i	-0.361-0.173i	-0.333+0.184i	0.52-0.027i	0.109-0.147i	-0.501+0.23i	0.186+0.222i
Y13	-0.366+0.091i	-0.408-0.09i	0.125+0.07i	0.266-0.098i	-0.241-0.081i	-0.171+0.09i	0.241-0.058i	0.02-0.035i	-0.32+0.225i	0.175+0.114i
Y14	-0.166+0.215i	-0.227+0.022i	0.052+0.047i	0.192-0.029i	-0.157+0.002i	-0.038+0.125i	0.119-0.117i	-0.043-0.167i	-0.196+0.081i	0.084-0.03i
Y15	0.473+0.007i	-0.054+0.033i	-0.049-0.056i	0.034-0.005i	-0.031+0.09i	0.04+0.084i	0.105-0.183i	-0.041-0.142i	-0.078+0.096i	0.07+0.004i
Y16	0.383-0.344i	-0.091i	-0.058-0.069i	-0.034-0.039i	0.004+0.088i	-0.01+0.006i	0.028-0.089i	-0.011+0.065i	-0.021+0.183i	0.086+0.029i
Y17	-0.586-0.076i	-0.138+0.115i	0.036+0.126i	0.09-0.075i	-0.05-0.024i	-0.042+0.005i	-0.021+0.04i	-0.018+0.085i	-0.012+0.056i	0.033-0.022i
Y18	-0.282+0.067i	-0.147+0.186i	0.118+0.227i	0.141-0.079i	-0.009-0.121i	-0.048+0.067i	0.019+0.03i	0.001-0.115i	-0.045-0.098i	-0.051+0.027i
Y19	-0.082+0.028i	-0.048+0.136i	0.04+0.053i	0.015-0.108i	-0.048-0.086i	-0.018+0.112i	0.103+0.054i	0.053-0.157i	-0.041-0.082i	-0.06+0.087i

Supplementary Note 1: Transmission bounds in conventional space-coiling metamaterials

The equivalent model of conventional space-coiling metamaterials is based on the classical three medium acoustic transmission in which the transmission coefficient can be derived as:

$$T=\frac{4}{(1+Z_2/Z_3+Z_1/Z_2+Z_1/Z_3)\ \mathrm{e}^{-ik_2l_2}+(1-Z_2/Z_3-Z_1/Z_2+Z_1/Z_3)\ \mathrm{e}^{ik_2l_2}} \tag{1}$$

In the case of the space-coiling metamaterial shown in Figure 1a, $l_2=n_\mathrm{r}t$, $Z_1=Z_3=\frac{\rho_1c_1}{a}$ and $Z_2=\frac{\rho_2c_2}{d}$. Substituting the above parameters into Supplementary Equation 1 and given that all media are composed of the same material, the transmission coefficient may be calculated as:

$$T=\frac{4}{\left(1+\frac{a}{d}\right)\left(1+\frac{d}{a}\right)\mathrm{e}^{-ik_0n_\mathrm{r}t}+\left(1-\frac{a}{d}\right)\left(1-\frac{d}{a}\right)\mathrm{e}^{ik_0n_\mathrm{r}t}} \tag{2}$$

Please note that throughout this paper, the $\mathrm{e}^{-i\omega t}$ convention has been considered and one may reach the complex conjugate forms of the equations by following the $\mathrm{e}^{i\omega t}$ convention.

In order to derive the relationship between phase and amplitude of the transmission coefficient, the denominator of Supplementary Equation 2 has initially been simplified to the trigonometric form and the amplitude is subsequently determined in terms of the transmission phase:

$$T=|T|\mathrm{e}^{i\theta}=\frac{1}{\cos(k_0n_\mathrm{r}t)-\frac{i}{2}(a/d+d/a)\sin(k_0n_\mathrm{r}t)} \tag{3}$$

From Supplementary Equation 3, the amplitude and phase can be derived as:

$$|T|=\sqrt{\frac{1}{\cos^2(k_0n_\mathrm{r}t)+\frac{1}{4}(a/d+d/a)^2\sin^2(k_0n_\mathrm{r}t)}}=\frac{1}{|\cos(k_0n_\mathrm{r}t)|}\sqrt{\frac{1}{1+\frac{1}{4}(a/d+d/a)^2\tan^2(k_0n_\mathrm{r}t)}} \tag{4}$$

$$\theta=\tan^{-1}\left(\frac{1}{2}\left(\frac{a}{d}+\frac{d}{a}\right)\tan(k_0n_\mathrm{r}t)\right)\xrightarrow{\text{yields}}\tan\theta=\frac{1}{2}\left(\frac{a}{d}+\frac{d}{a}\right)\tan(k_0n_\mathrm{r}t) \tag{5}$$

From Supplementary Equation 5, one can conclude that:

$$\frac{1}{|\cos(k_0n_\mathrm{r}t)|}=\sqrt{1+\frac{\tan^2\theta}{\frac{1}{4}\left(\frac{a}{d}+\frac{d}{a}\right)^2}} \tag{6}$$

Defining $S = \frac{1}{2}\left(\frac{a}{d} + \frac{d}{a}\right)$ and employing Supplementary Equation 4-6, the transmission amplitude in terms of the transmission phase is as follows:

$$|T| = \sqrt{\frac{1+\frac{\tan^2(\theta)}{S^2}}{1+\tan^2(\theta)}} \tag{7}$$

Supplementary Note 2: Transfer matrix method to derive transmission coefficient for space-coiling metamaterials

In this section, the transmission through the space-coiling structure has been derived analytically, considering the complex internal geometry (Supplementary Figure 1a). To this end, the transfer matrix method has been utilized to extract the governing relationship and correlate input pressure and velocity to the output condition. Considering a single coil of a unit cell (Supplementary Figure 1b) of the space-coiling metamaterial, one may derive the propagation tensor that relates the output condition to the input condition, which can be written in the form of:

$$\begin{bmatrix} P_2 \\ U_2 \end{bmatrix} = [M_1]\begin{bmatrix} P_1 \\ U_1 \end{bmatrix} \tag{8}$$

Eventually, total transmission through the space-coiling structure (Supplementary Figure 1a) may be derived as below:

$$\begin{bmatrix} P_{\text{out}} \\ U_{\text{out}} \end{bmatrix} = [M_N] \ldots . [M_2][M_1]\begin{bmatrix} P_{\text{in}} \\ U_{\text{in}} \end{bmatrix} \tag{9}$$

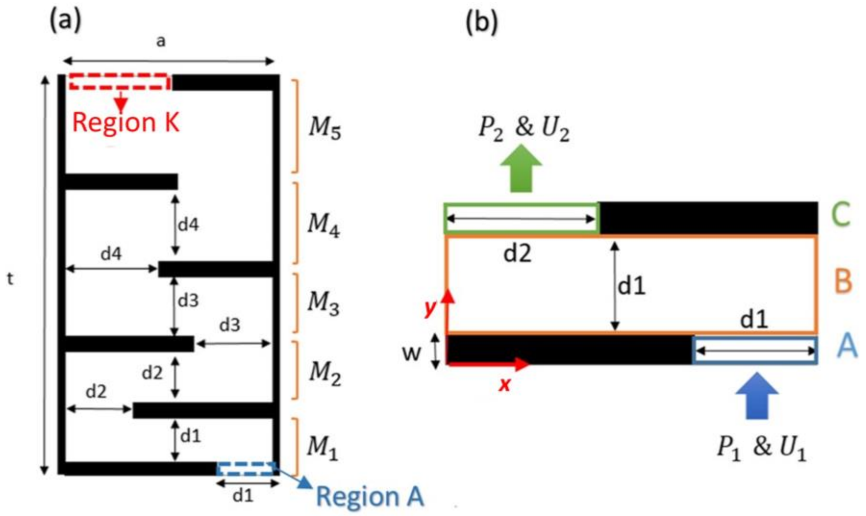

Supplementary Figure 1. Illustration of internal structure of space–coiling unit cell. (a) Space-coiling structure associated propagation tensor for each section. (b) Single coil structure, which has been divided into three sections: section A is the input port, section B is the main channel and section C is the output port.

Please note that for conventional space-coiling metamaterials in which the channel width is uniform throughout the unit cell, all propagation tensors (Ms) would be identical and this would represent a unique case. However, in the case of the gradient space-coiling metamaterials introduced in this work, the use of the transfer matrix method remains applicable, though due to the change in geometry from coil to coil, the propagation tensor would not necessarily remain identical.

In order to determine the propagation tensor in a single coil, the region has been divided into the three sections shown in Supplementary Figure 1b. Pressure and velocity in each region can be written using modal superposition in the rectangular waveguide, however, since regions A and C have relatively small dimensions when compared to the wavelength, only the principal mode is considered herein for these two regions. Pressure and velocity in these regions are as follow:

A: $\begin{cases} P(y) = A_1 e^{iky} + A_2 e^{-ik(y-w)} & (10) \\ U(y) = \frac{1}{\rho c}\left[A_1 e^{iky} - A_2 e^{-ik(y-w)}\right] & (11) \end{cases}$

C: $\begin{cases} P(y) = C_1 e^{ik(y-w-d1)} + C_2 e^{-ik(y-2w-d1)} & (12) \\ U(y) = \frac{1}{\rho c}\left[C_1 e^{ik(y-w-d1)} - C_2 e^{-ik(y-2w-d1)}\right] & (13) \end{cases}$

B: $\begin{cases} P(x,y) = \sum_{n=0}^{\infty} \cos(a_n x)\left[B_{1n} e^{ik_n (y-w)} + B_{2n} e^{-ik_n (y-w-d1)}\right] & (14) \\ U(x,y) = \sum_{n=0}^{\infty} \frac{k_n}{\rho c k} \cos(a_n x)\left[B_{1n} e^{ik_n (y-w)} - B_{2n} e^{-ik_n (y-w-d1)}\right] & (15) \end{cases}$

in which wave number is defined as $k = \frac{\omega}{c}$ and the *n*th eigenmode and wavenumber in region B can be derived as $a_n = \frac{n\pi}{a}$, $k_n = \sqrt{k^2 - a_n^2}$. The geometrical parameters *a*, *w* and *d1* are shown in Supplementary Figure 1b. Given the above equations, one may conclude that the problem of deriving Supplementary Equation 8 for a single coil can be reduced to deriving the relationship between the coefficient of A and C in which A_1 and C_1 are forward traveling wave coefficients and A_2 and C_2 are backward traveling wave coefficients as follows:

$$\begin{bmatrix} C_1 \\ C_2 \end{bmatrix} = [T_1] \begin{bmatrix} A_1 \\ A_2 \end{bmatrix} \quad (16)$$

$$\begin{bmatrix} K_1 \\ K_2 \end{bmatrix} = [T_N] \ldots . [T_2][T_1] \begin{bmatrix} A_1 \\ A_2 \end{bmatrix} \quad (17)$$

in which the forward traveling wave coefficient K_1 and the backward traveling wave coefficient K_2 correspond to the output port of the final coil shown in Supplementary Figure 1a.

Next, pressure and velocity boundary conditions at *y* = *w* and *y* = *d1*+*w* have been applied.

At: $y = w\,,\ \ a - d1 \le x \le a$

$$A_1 e^{ikw} + A_2 = \sum_{n=0}^{\infty} \cos(a_n x)\left[B_{1n} + B_{2n} e^{ik_n d1}\right] \quad (18)$$

$$\left[A_1 e^{ikw} - A_2\right] = \sum_{n=0}^{\infty} \frac{a k_n}{d_1 k} \cos(a_n x)\left[B_{1n} - B_{2n} e^{ik_n d1}\right] \quad (19)$$

At: $y = w + d1\,,\ \ 0 \le x \le d2$

$$C_1 + C_2 e^{ikw} = \sum_{n=0}^{\infty} \cos(a_n x) \left[B_{1n} e^{ik_n\, d1} + B_{2n}\right] \quad (20)$$

$$\left[C_1 - C_2 e^{ikw}\right] = \sum_{n=0}^{\infty} \frac{ak_n}{d_2 k} \cos(a_n x) \left[B_{1n} e^{ik_n\, d1} - B_{2n}\right] \quad (21)$$

In order to ensure that Supplementary Equations 18-21 are a solvable set of equations to relate the coefficients in Region A (A_1 and A_2) and Region C (C_1 and C_2), one needs to relieve the *x*-dependency, along with deriving the nth mode's coefficient in Region B. Thus, the problem has been simplified by first multiplying the velocity boundary conditions (Supplementary Equation 19) and (Supplementary Equations 21) by the factor of $\cos(a_m x)$ and taking the normalized line integral with respect to *x* from both sides of Supplementary Equations 18-21. Given the orthogonality of the trigonometric functions, the summation terms in the right-hand-side of Supplementary Equation 19 and Supplementary Equation 21 will vanish, save for the case when *m* = *n*. Eventually, the simplified form of the boundary conditions yields the following:

At: $y = w$

$$A_1 e^{ikw} + A_2 = \sum_{n=0}^{\infty} \frac{1}{d1} \int_{a-d1}^{a} \cos(a_n x)\, dx \left[B_{1n} + B_{2n} e^{ik_n\, d1}\right] \quad (22)$$

$$\left[A_1 e^{ikw} - A_2\right] \frac{1}{d1} \int_{a-d1}^{a} \cos(a_n x)\, dx = \frac{ak_n}{\epsilon_n d_1 k} \left[B_{1n} - B_{2n} e^{ik_n\, d1}\right] \quad (23)$$

At: $y = w + d1$

$$C_1 + C_2 e^{ikw} = \sum_{n=0}^{\infty} \frac{1}{d2} \int_{0}^{d2} \cos(a_n x)\, dx \left[B_{1n} e^{ik_n\, d1} + B_{2n}\right] \quad (24)$$

$$\left[C_1 - C_2 e^{ikw}\right] \frac{1}{d2} \int_{0}^{d2} \cos(a_n x)\, dx = \frac{ak_n}{\epsilon_n d_2 k} \left[B_{1n} e^{ik_n\, d1} - B_{2n}\right] \quad (25)$$

in which Neumann Factor, ϵ_n ,is defined as $\epsilon_n = 2$ for $n \neq 0$ and has the value of $\epsilon_n = 1$ if $n = 0$.

Using Supplementary Equation 23 and Supplementary Equation 25, B_{1n} & B_{2n} can be derived in terms of *A*'s and *C*'s coefficients and, consequently, by substituting in Supplementary Equation

22 and Supplementary Equation 25, the relationship between *A*'s and *C*'s coefficients are thusly determined:

$$\begin{bmatrix} C_1 \\ C_2 \end{bmatrix} = \ [T_1] \begin{bmatrix} A_1 \\ A_2 \end{bmatrix} \rightarrow \begin{bmatrix} C_1 \\ C_2 \end{bmatrix} = \begin{bmatrix} T_{11} & T_{12} \\ T_{21} & T_{22} \end{bmatrix} \begin{bmatrix} A_1 \\ A_2 \end{bmatrix} \tag{26}$$

$$T_{11} = \frac{\mathrm{e}^{ikw}(\gamma_3\gamma_1+\gamma_2)}{(1+\gamma_1)} \tag{27}$$

$$T_{12} = \frac{(\gamma_4\gamma_1-\gamma_2)}{(1+\gamma_1)} \tag{28}$$

$$T_{21} = \frac{(\gamma_2-\gamma_3)}{(1+\gamma_1)} \tag{29}$$

$$T_{22} = \frac{-(\gamma_4+\gamma_2)}{\mathrm{e}^{ikw}(1+\gamma_1)} \tag{30}$$

in which γ_1, γ_2, γ_3 and γ_4 are derived as follows:

$$\gamma_1 = \frac{1+\left[\sum_{n=0}^{\infty}\frac{\varphi_n^2}{d_2}\left(\frac{2\epsilon_n k\varphi_n^2\ \left(\mathrm{e}^{2ik_n d_1}+1\right)}{ak_n\left(\mathrm{e}^{2ik_n d_1}-1\right)}\right)\right]}{1-\left[\sum_{n=0}^{\infty}\frac{\varphi_n^2}{d_2}\left(\frac{2\epsilon_n k\varphi_n^2\ \left(\mathrm{e}^{2ik_n d_1}+1\right)}{ak_n\left(\mathrm{e}^{2ik_n d_1}-1\right)}\right)\right]} \tag{31}$$

$$\gamma_2 = \frac{\left[\sum_{n=0}^{\infty}\frac{\varphi_n^2}{d_2}\left(\frac{4\,\epsilon_n k\varphi_n^1\ \left(\mathrm{e}^{ik_n d_1}\right)}{ak_n\left(\mathrm{e}^{2ik_n d_1}-1\right)}\right)\right]}{\left[\sum_{n=0}^{\infty}\frac{\varphi_n^2}{d_2}\left(\frac{2\epsilon_n k\varphi_n^2\ \left(\mathrm{e}^{2ik_n d_1}+1\right)}{ak_n\left(\mathrm{e}^{2ik_n d_1}-1\right)}\right)\right]-1} \tag{32}$$

$$\gamma_3 = \frac{1+\left[\sum_{n=0}^{\infty}\frac{\varphi_n^1}{d_1}\left(\frac{2\,\epsilon_n k\varphi_n^1\ \left(\mathrm{e}^{2ik_n d_1}+1\right)}{ak_n\left(\mathrm{e}^{2ik_n d_1}-1\right)}\right)\right]}{\left[\sum_{n=0}^{\infty}\frac{\varphi_n^1}{d_1}\left(\frac{4\epsilon_n k\varphi_n^2\ \left(\mathrm{e}^{ik_n d_1}\right)}{ak_n\left(\mathrm{e}^{2ik_n d_1}-1\right)}\right)\right]} \tag{33}$$

$$\gamma_4 = \frac{1-\left[\sum_{n=0}^{\infty}\frac{\varphi_n^1}{d_1}\left(\frac{2\,\epsilon_n k\varphi_n^1\ \left(\mathrm{e}^{2ik_n d_1}+1\right)}{ak_n\left(\mathrm{e}^{2ik_n d_1}-1\right)}\right)\right]}{\left[\sum_{n=0}^{\infty}\frac{\varphi_n^1}{d_1}\left(\frac{4\epsilon_n k\varphi_n^2\ \left(\mathrm{e}^{ik_n d_1}\right)}{ak_n\left(\mathrm{e}^{2ik_n d_1}-1\right)}\right)\right]} \tag{34}$$

In Supplementary Equations 31-34, φ_n^1 and φ_n^2 are defined as follows:

$$\varphi_n^1 \ = \int_{a-d1}^{a} \cos(a_n x)\, dx \tag{35}$$

$$\varphi_n^2 \ = \int_0^{d2} \cos(a_n x)\, dx \tag{36}$$

Employing Supplementary Equations 26-36, the propagation tensor for a single coil of a unit cell (Supplementary Equation 16) can be calculated. By taking into account the effects of all coils in the space-coiling metamaterial, the propagation tensor correlating the last output port (output of the entire unit cell) to the coefficients of the first input port (input of the entire unit cell) can be derived using Supplementary Equation 17. Finally, employing the resultant propagation tensor of the space-coiling metamaterial, the transmission coefficient is calculated.

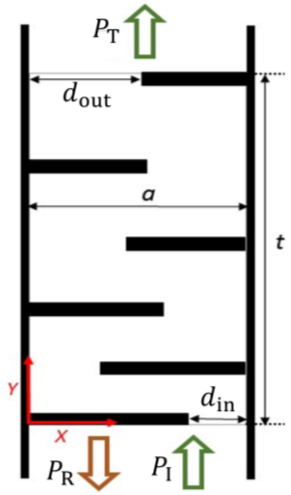

Supplementary Figure 2. Transmission and reflection from space-coiling unit cell.

Pressure and velocity boundary condition at *y* = 0 (Supplementary Figure 2):

$$P_\mathrm{I} + P_\mathrm{R} = A_1 + A_2 \mathrm{e}^{ikw} \tag{37}$$

$$P_\mathrm{I} - P_\mathrm{R} = \frac{d_\mathrm{in}}{a}(A_1 - A_2 \mathrm{e}^{ikw}) \tag{38}$$

Pressure and velocity boundary condition at *y* = *t*:

$$P_\mathrm{T} = K_1 \mathrm{e}^{ikw} + K_2 \tag{39}$$

$$P_\mathrm{T} = \frac{d_\mathrm{out}}{a}(K_1 \mathrm{e}^{ikw} - K_2) \tag{40}$$

Additionally, using the propagation tensor derived for the space-coiling structure, *K*'s coefficient can be defined in terms of *A*'s coefficients.

$$\begin{bmatrix} K_1 \\ K_2 \end{bmatrix} = \begin{bmatrix} M_{11} & M_{12} \\ M_{21} & M_{22} \end{bmatrix} \begin{bmatrix} A_1 \\ A_2 \end{bmatrix} \tag{41}$$

Using Supplementary Equations 37-41), the complex transmission ($T = \frac{P_\mathrm{T}}{P_\mathrm{I}}$) and reflection ($R = \frac{P_\mathrm{R}}{P_\mathrm{I}}$) coefficient are determined.

$$T = \frac{1}{2}\left[\left(\frac{a}{d_\mathrm{in}} + 1\right) + \left(1 - \frac{a}{d_\mathrm{in}}\right) R\right]\left(M_{11}\mathrm{e}^{ikw} + M_{21}\right) + \frac{1}{2}\left[\left(\frac{a}{d_\mathrm{in}} + 1\right) R + \left(1 - \frac{a}{d_\mathrm{in}}\right)\right]\left(M_{12} + M_{22}\mathrm{e}^{-ikw}\right) \tag{42}$$

$$R = \frac{\eta\left(\frac{a}{d_\mathrm{in}} - 1\right) - \left(1 + \frac{a}{d_\mathrm{in}}\right)}{\eta\left(\frac{a}{d_\mathrm{in}} + 1\right) + \left(1 - \frac{a}{d_\mathrm{in}}\right)} \tag{43}$$

in which η is defined as:

$$\eta = \frac{M_{12}\left(\frac{d_\mathrm{out}}{a} - 1\right) - M_{22}\mathrm{e}^{-ikw}\left(\frac{d_\mathrm{out}}{a} + 1\right)}{M_{11}\mathrm{e}^{ikw}\left(\frac{d_\mathrm{out}}{a} - 1\right) - M_{21}\left(\frac{d_\mathrm{out}}{a} + 1\right)} \tag{44}$$

In order to validate the analytical solution, the solution result has been compared with a numerical model of a single unit cell constructed using COMSOL Multiphysics software. The unit cell modeled herein, with background medium of air, features a channel width of *d* = 2cm and is composed of 20 coils (*N* = 20) with overall dimensions of 55 x 8.5 cm. By sweeping the frequency, the resultant transmission and reflection amplitude and phase have been obtained using both numerical (COMSOL) and analytical models (transfer matrix method) discussed herein, with the results shown in Supplementary Figure 3. The results achieved using both the numerical and analytical approaches are in a high degree of agreement, thereby validating the applicability of the presented model for studying the space-coiling metamaterial behavior.

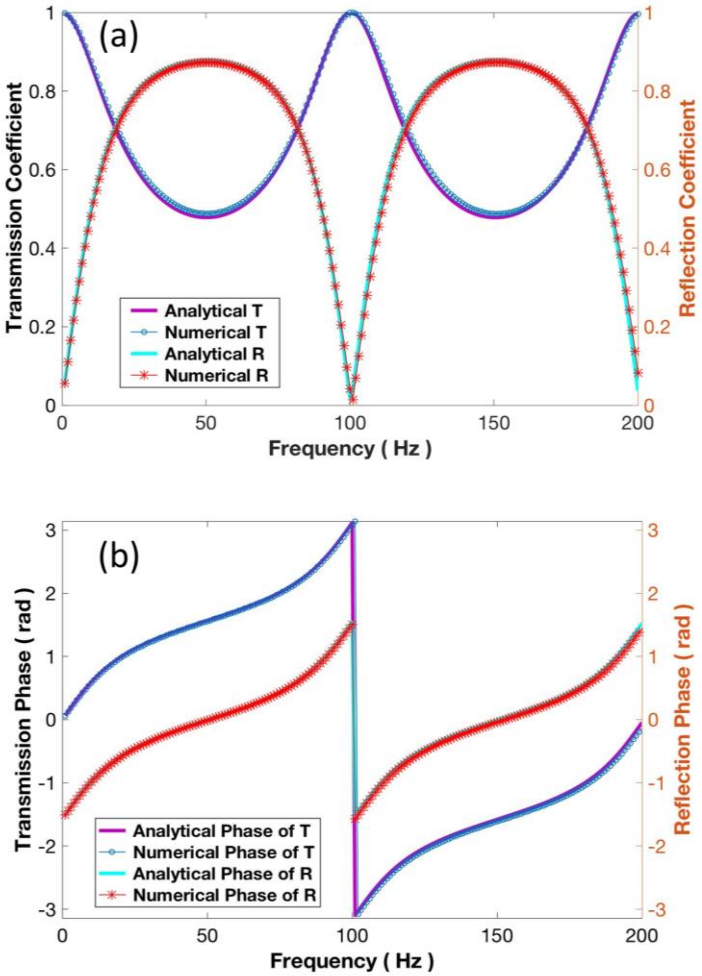

Supplementary Figure 3. Comparison between analytical and numerical transmission and reflection. (a) Transmission and reflection amplitude. (b) Transmission and reflection phase.

Supplementary Note 3: Horn-like space-coiling metamaterials

The behavior of horn-like space-coiling metamaterials may be analyzed using the analytical approach presented in Supplementary Note 2, in which in Supplementary Equation 17, each propagation tensors (T_i) will be computed based on the associated coil's geometry. However, due to the complexity of the aforementioned analytical solution, the use of an equivalent model to formulate the performance of the structure (similar to Supplementary Equation 2) will be beneficial for markedly simplifying the incorporation of this structure in various applications. In this section,

the horn-like space-coiling structure has been approximated with the equivalent horn and, subsequently, the transmission coefficient based on the horn model has been derived.

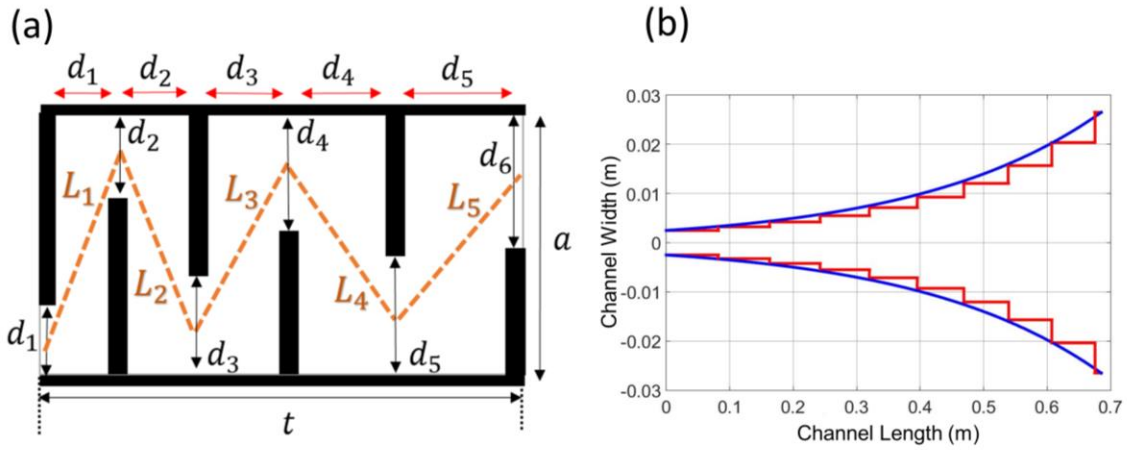

Supplementary Figure 4. Space-coiling unit cell with gradual change in channel width as an exponential horn. (a) Horn-like space-coiling metamaterial with effective length of acoustic pathway shown with orange dashed-line. (b) Comparison between exact profile of the channel and approximated horn model.

The structure of the horn-like space-coiling metamaterial is shown in Supplementary Figure 4a. In this structure, the effective length of the acoustic pathway can be approximated with:

$$L_{\text{eff}} = \sum_{b=1}^{b=N} L_b \tag{45}$$

$$L_b = \sqrt{(a - d_b)^2 + (w + d_b)^2} \tag{46}$$

in which *N* is the number of coils (*N* = 5 in the structure depicted in Supplementary Figure 4a) and *w* is the wall thickness (see Supplementary Figure 1b). By calculating d_b and L_b for each coil, the acoustic pathway profile through the horn-like space-coiling metamaterial has been illustrated for one arbitrary structure (*N* = 10, *w* = 0.01m, *d*1 = 5mm, *a* = 8.5cm and CR = 1.3), shown in

Supplementary Figure 4b in red. At each section of the pathway, the profile has a width of d_b and a length of L_b, both of which change from one coil of a given unit cell to another.

The approximated horn-like profile is based on the assumption that the change in L_b values are small ((*a*) is dominant term in Supplementary Equation 46) and, consequently, channel width follows the below equation:

$$d = d_1 \mathrm{CR}^{\left(\frac{xN}{L_{\mathrm{eff}}}\right)} \tag{47}$$

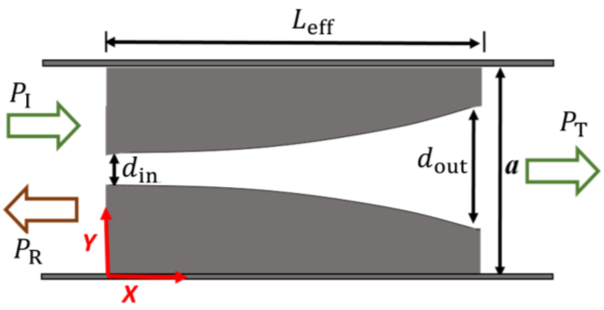

Supplementary Figure 5. Transmission through horn-like model.

The horn-like profile based on Supplementary Equation 47 is depicted in Supplementary Figure 4b (blue line) which demonstrates a good approximation of the exact profile (in red). By employing Supplementary Equation 47, the flare constant (*m*) of the approximated horn-like model can be derived as:

$$m = \frac{\partial}{\partial x}(\log(d)) \;\rightarrow\; m = \frac{N}{L_{\mathrm{eff}}}\log(\mathrm{CR}) \tag{48}$$

Next, using the equivalent model, the transmission coefficient has been calculated for the horn-like space-coiling metamaterial.

Using the Webster-horn equation for velocity potential, defined as:

$$\left(\frac{\partial^2}{\partial x^2} + m\frac{\partial}{\partial x} + k^2\right)\emptyset = 0 \tag{49}$$

where ∅ is the velocity potential, *m* is the flare constant, and *k* is the wave number. The solution of Supplementary Equation 49 has the form of:

$$\emptyset = C_1 \mathrm{e}^{\mu_1 x} + C_2 \mathrm{e}^{\mu_2 x} \tag{50}$$

$$\mu_1 = -\frac{m}{2} + \frac{i}{2}\sqrt{4k^2 - m^2} \tag{51}$$

$$\mu_2 = -\frac{m}{2} - \frac{i}{2}\sqrt{4k^2 - m^2} \tag{52}$$

From the velocity potential obtained in Supplementary Equation 50, one can calculate the pressure and velocity as:

$$V = \frac{\partial \emptyset}{\partial x} = C_1 \mu_1 \mathrm{e}^{\mu_1 x} + C_2 \mu_2 \mathrm{e}^{\mu_2 x} \tag{53}$$

$$P = -\rho \frac{\partial \emptyset}{\partial t} = -i\rho\omega(C_1 \mathrm{e}^{\mu_1 x} + C_2 \mathrm{e}^{\mu_2 x}) \tag{54}$$

Next, the pressure and velocity boundary conditions have been written at x=0 and x= L_{eff}.

At $x = 0$:

$$-i\rho\omega(C_1 + C_2) = P_{\mathrm{I}} + P_{\mathrm{R}} \tag{55}$$

$$C_1\mu_1 + C_2\mu_2 = \frac{a}{\rho c\, d_{\mathrm{in}}}(P_{\mathrm{I}} - P_{\mathrm{R}}) \tag{56}$$

At $x = L_{\mathrm{eff}}$:

$$-i\rho\omega(C_1 \mathrm{e}^{\mu_1 L_{\mathrm{eff}}} + C_2 \mathrm{e}^{\mu_2 L_{\mathrm{eff}}}) = P_{\mathrm{T}} \tag{57}$$

$$C_1\mu_1 \mathrm{e}^{\mu_1 L_{\mathrm{eff}}} + C_2\mu_2 \mathrm{e}^{\mu_2 L_{\mathrm{eff}}} = \frac{a}{\rho c\, d_{\mathrm{out}}}(P_{\mathrm{T}}) \tag{58}$$

From Supplementary Equations 55-58, the complex transmission coefficient has been derived.

$$T = |T|\mathrm{e}^{i\theta} = \frac{4}{\left(-\frac{k}{\beta}\frac{a}{d_{\mathrm{out}}}+1+\frac{d_{\mathrm{in}}}{d_{\mathrm{out}}}-\frac{k}{\beta}\frac{d_{\mathrm{in}}}{a}+i\frac{\gamma}{\beta}(1-\frac{d_{\mathrm{in}}}{d_{\mathrm{out}}})\right)\mathrm{e}^{(\gamma+i\beta)L_{\mathrm{eff}}}+\left(\frac{k}{\beta}\frac{a}{d_{\mathrm{out}}}+1+\frac{d_{\mathrm{in}}}{d_{\mathrm{out}}}+\frac{k}{\beta}\frac{d_{\mathrm{in}}}{a}-i\frac{\gamma}{\beta}(1-\frac{d_{\mathrm{in}}}{d_{\mathrm{out}}})\right)\mathrm{e}^{(\gamma-i\beta)L_{\mathrm{eff}}}} \tag{59}$$

where d_{in} and d_{out} are the channel width at the input and output ports, respectively, a is unit cell width (all are shown in Supplementary Figure 5), *k* is wave number, and γ and β are defined as:

$$\gamma = \frac{m}{2} \tag{60}$$

$$\beta = \frac{1}{2}\sqrt{4k^2 - m^2} \tag{61}$$

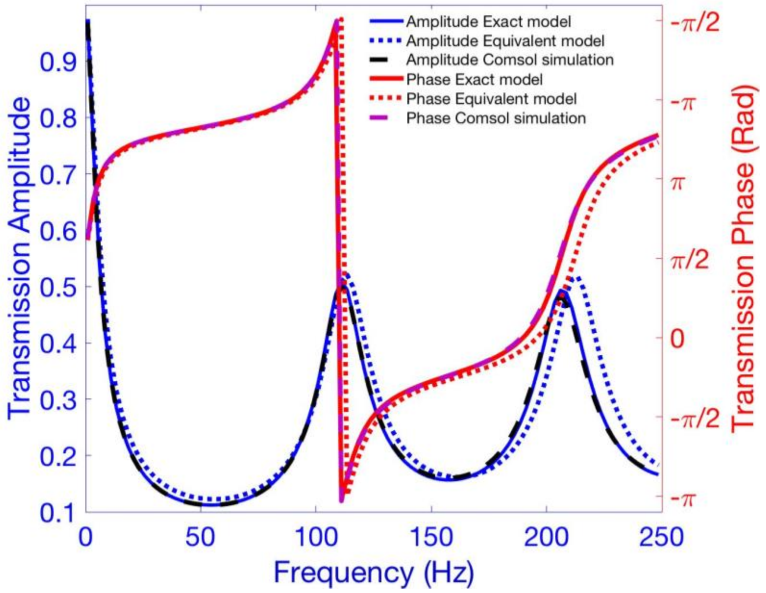

Supplementary Figure 6. Transmission through horn-like space-coiling metamaterial derived from three different approaches. Exact model refers to the analytical solution presented in Supplementary Note 2 and equivalent model refers to the horn-like model discussed in Supplementary Note 3.

In order to validate the applicability of the presented equivalent model for the horn-like space-coiling structure, the results from the equivalent model have been compared with both a COMSOL numerical model and the exact analytical solution (transfer matrix method) presented in Supplementary Note 2. The structure considered herein has the following parameters: N = 14, w = 1cm, d_1 = 3mm, a = 8.5cm and CR = 1.2. The comparisons of the equivalent model with both the COMSOL model and the exact solution are shown in Supplementary Figure 6. From the results shown in Supplementary Figure 6, it can be observed that the numerical results obtained from the simulated model in COMSOL Multiphysics are in precise agreement with the results of

the analytical approach discussed in Supplementary Note 2 (labeled herein as the exact model). Moreover, the results from the equivalent horn-like model are in good agreement, with only small deviations from the exact model and the numerical results. This small discrepancy is largely due to the simple approximation of L_{eff} and can be improved with more exacting computation of this parameter. The presented equivalent model drastically simplifies the design of the Horn-like space coiling-metamaterial and readily provides an initial good estimate of structural performance for further experimental or numerical validation. Finally, using the equivalent horn-like model, the transmission bound similar to the one derived for conventional space-coiling metamaterials has been derived.

From Supplementary Equation 59 and given the relation that $\gamma^2 + \beta^2 = k^2$, the transmission phase and amplitude can be derived as:

$$\theta = \tan^{-1}\left[\frac{k\left(\frac{a}{d_{\text{out}}}+\frac{d_{\text{in}}}{a}\right)\sin(\beta L_{\text{eff}})}{\beta\left(1+\frac{d_{\text{in}}}{d_{\text{out}}}\right)\cos(\beta L_{\text{eff}})+\gamma\left(1-\frac{d_{\text{in}}}{d_{\text{out}}}\right)\sin(\beta L_{\text{eff}})}\right] \tag{62}$$

$$|T| = 2\mathrm{e}^{-\gamma L_{\text{eff}}}\sqrt{\frac{\frac{1}{(1+d_{\text{in}}/d_{\text{out}})^2}+\frac{\tan^2\theta}{(d_{\text{in}}/a+a/d_{\text{out}})^2}-\frac{4\gamma^2 d_{\text{in}}\tan^2\theta}{k^2 d_{\text{out}}(d_{\text{in}}/a+a/d_{\text{out}})^2(1+d_{\text{in}}/d_{\text{out}})^2}+\frac{2\gamma\,(1-d_{\text{in}}/d_{\text{out}})\tan\theta}{k(d_{\text{in}}/a+a/d_{\text{out}})^2(1+d_{\text{in}}/d_{\text{out}})^2}}{1+\tan^2\theta}} \tag{63}$$

In the case of a structure with small CR and, consequently small flare constant of a frequency higher than that of the equivalent horn cut-off frequency, the ratio $\frac{\gamma}{k}$ would be small and has been omitted to simplify the equation.

Thus, Supplementary Equation 63 yields the form of:

$$|T| = \frac{2e^{-\gamma L_{\text{eff}}}}{(1+\frac{d_{\text{in}}}{d_{\text{out}}})}\sqrt{\frac{1+\frac{\tan^2(\theta)}{S^2}}{1+\tan^2(\theta)}} \tag{64}$$

in which *S* is defined as:

$$S = \frac{(\frac{a}{d_{\text{out}}}+\frac{d_{\text{in}}}{a})}{(1+\frac{d_{\text{in}}}{d_{\text{out}}})} \quad (65)$$

By substituting $\gamma = \frac{m}{2} = \frac{N}{2L_{\text{eff}}}\log(\text{CR})$, along with the fact that $\frac{d_{\text{in}}}{d_{\text{out}}} = \text{CR}^{-N}$, the transmission amplitude can be written as:

$$|T| = \frac{2}{\text{CR}^{N/2}+\text{CR}^{-N/2}}\sqrt{\frac{1+\frac{\tan^2(\theta)}{S^2}}{1+\tan^2(\theta)}} \quad (66)$$

Supplementary Note 4: Metasurface for full wavefront manipulation

Unit cell width as a critical design parameter. While phase-based wavefront modulation relies on an optimization procedure to tune the transmitted phase in each unit cell, full wave modulation (phase-amplitude) benefits from the use of the phase-conjugation method to tune the phase and amplitude of transmission at each unit cell. In another words, in full wave modulation, the complex transmission is obtained by discretizing the conjugated wave profile along the metasurface based on the width and the number of present unit cells. Consequently, for a given length of the metasurface, the unit cell's width is a critical design parameter, which can directly affect the

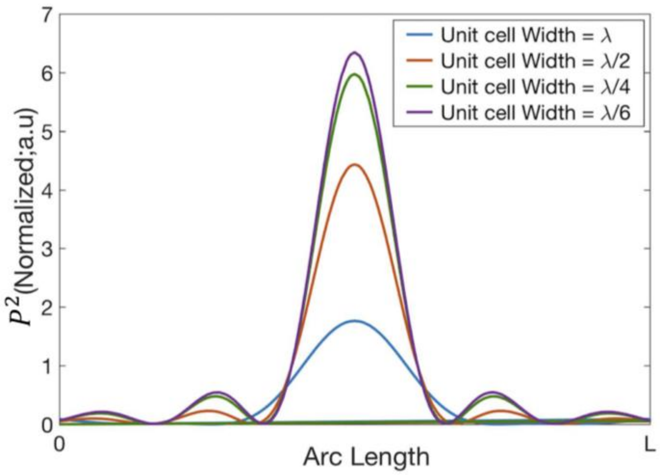

Supplementary Figure 7. Sound focusing profiles as a function of unit cell width.

conversion output. Smaller unit cell's widths provide a finer discretization and, consequently, near-ideal wavefront shaping capability. As an example, the sound focusing case discussed in this work has been considered and the focusing performance for different values of unit cell width have been analyzed to yield insight into the effect of this key parameter. The sound focusing profile, similar to the Figure 5c, is shown in Supplementary Figure 7 for four different values of unit cell width ranging from λ to $\lambda/6$. Supplementary Figure 7 clearly demonstrates that the unit cell's width plays a critical role in successful full wave modulation and smaller widths are preferable in order to obtain near-ideal wavefront conversion.

Comparison of phase and phase-amplitude modulation. While phase modulation represents a highly effective method for wavefront shaping, the additional degree of freedom afforded by full wave modulation (phase-amplitude) may yield improved performance and capability. As an illustrative example, in the case of acoustic beam splitting into π/12 and - π/12 directions, both phase modulation and phase-amplitude modulation have been considered. In both cases, a metasurface with length of 5λ and composed of 30 unit cells has been assumed (vertical black line on the left side of Supplementary Figures 8a and 8b). In the case of phase modulation, given the desired directions of the split beams, the ideal phase at each unit cell has been obtained using

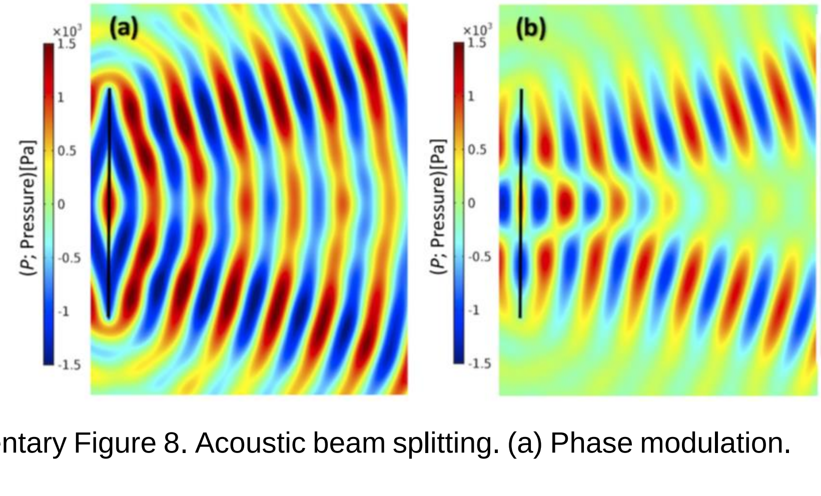

Supplementary Figure 8. Acoustic beam splitting. (a) Phase modulation. (b) Phase–amplitude modulation.

the generalized Snell's law. Considering unity transmission amplitude along with the transmission phase found from the generalized Snell's law at each unit cell, the resulting waveform, representing the ideal case, is shown in Supplementary Figure 8a. For comparison, a similar metasurface has been considered for phase-amplitude modulation, in which using the phase-conjugation method, the ideal transmission phase and amplitude at each unit cell have been assigned with the resulting wavefront shown in Supplementary Figure 8b. Comparing the results shown in Supplementary Figures 8a and 8b, it may be readily observed that a markedly improved pressure uniformity is achieved using phase-amplitude modulation when compared to phase modulation. Using phase-amplitude modulation, a clear pressure resolution between the acoustic beams may be achieved, while such a clear pressure resolution has not been obtained with phase modulation.

Geometrical features of the designed metasurface. The geometrical characteristics of each unit cell are listed in Supplementary Table 1. Please note that the metasurface is symmetric with

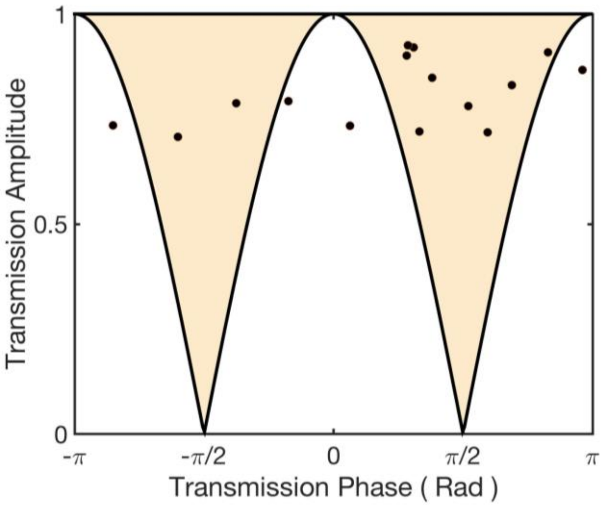

Supplementary Figure 9. Set of transmission values in the case of acoustic focusing from time-reversal method shown in black dots. The accessible region of the phase-amplitude in conventional space-coiling metamaterials is highlighted by the yellow regions.

respect to the *x*-axis and unit cells 1 to 15 are identical to unit cells 30-16. The targeted and ideal transmission phase and amplitude values obtained from the time-reversal technique are listed for each unit cell and the achieved phase and amplitude with both the numerical approach and the analytical transfer matrix method are shown in Supplementary Table 1.

Please note that the resultant transmission phase and amplitude from the time-reversal method are not placed within the accessible region of the complex transmission in conventional space-coiling metamaterials. For instance, transmission values in the case of acoustic focusing from the time-reversal method are plotted in the phase-amplitude chart shown in Supplementary Figure 9 in which the yellow region demonstrates the accessible transmission region of conventional space-coiling metamaterials. Data points outside this region are highlighted in the Supplementary Table 1, which have been achieved by the horn-like space-coiling structure. The results shown herein demonstrate that by incorporating the horn-like space-coiling structure, the phase and amplitude of the transmission coefficient may be simultaneously modulated, which offers the opportunity for complete wavefront manipulation.
